# Protein-Signaled Guided Bone Regeneration Using Titanium Mesh and Rh-BMP2 in Oral Surgery: A Case Report Involving Left Mandibular Reconstruction after Tumor Resection

**DOI:** 10.2174/1874210601206010051

**Published:** 2012-03-09

**Authors:** M Cicciù, A.S Herford, E Stoffella, G Cervino, D Cicciù

**Affiliations:** 1 Human Pathology Department, University of Messina University of Messina School of Dentistry; 2 Oral and Maxillofacial Surgery Loma Linda University, Loma Linda, CA, USA; 3 Linda University, Loma Linda, CA, US; 4 Department of Odontostomatology University of Messina School of Dentistry; 5 Department of Odontostomatology University of Messina School of Dentistry

**Keywords:** BMP2 human protein, bone reconstruction, bone regeneration, oral surgery, titanium mesh.

## Abstract

Recombinant human bone morphogenetic protein-2 (rhBMP-2) is an osteoinductive protein approved for use in oral and maxillofacial defect reconstruction. Growth factors act as mediators of cellular growth on morphogenesis and mythogenesis phases. Utilized as recombinant proteins, these growth factors need the presence of local target cells capable of obtaining the required results. This cell population may be present at the wound site or added to scaffolding material before implantation at the surgical site.

The aim of this study is to evaluate the clinical and radiographic results of a reported case with a large bone defect, treated with an absorbable collagen sponge, rhBMP-2 and a titanium plate and mesh. The Authors want to report a case which shows the resulting effectiveness of the rhBMP2 action regarding a large, mandibular defect reconstruction. This case also shows how the removal of a rare tumor such as a ghost cell tumor of the jaw may be treated without harvesting bone from another body site. A quick diagnosis of the lesions is important in order to perform the most suitable treatment. The Authors also underline the clinical and histological steps to insure the correct treatment is carried out to solve the case.

Moreover, from results obtained from this case, it is possible to highlight several clinical benefits for the patient by adding rhBMP-2 to the common allograft to not only have alveolar reconstruction defects and sinus floor augmentation, but also to have alveolar cleft reconstruction and to treat segmental defects.

## INTRODUCTION

Recombinant human bone morphogenetic protein-2 (rhBMP-2) is an osteoinductive growth factor that has the potential to greatly reduce the need for autogenous bone grafts [1]. Urist demonstrated the capabilities of demineralized bone matrixes to induce ectopic bone formation in a rat muscle pouch. He first introduced the concept that growth factors can induce bone formation independently of the bone tissue condition [2]. 

The emergence of rhBMP-2 as a viable alternative to common bone grafts is related to two important clinical challenges. The first is to eliminate the need to harvest bone from the iliac crest or other sites when performing oral and maxillofacial reconstruction techniques because of morbidity associated with these procedures [3]. The second reason is to enhance the degree of new bone formation, ultimately leading to positioning of dental implants, for treating several defects of the facial skeleton [4, 5].

Autogenous bone has long been considered the “gold standard”. However, there are significant, potential disadvantages. Probably the greatest potential advantage for using rhBMP-2 is avoiding the need for graft harvesting thus eliminating any morbidity associated with it. Complications of harvesting extra oral bone grafts such as the iliac crest may occur in as many as 15% to 25% of patients [6-9]. 

Problems associated with iliac crest bone harvesting are pain and the risk of significant morbidity. Graft harvesting complications include increased surgical morbidity from an additional operative site, including chronic donor site pain, increased operative time, and additional cost. For larger defects, the quantity of bone available for harvest may be insufficient for larger defects or in patients who have undergone previous graft harvests. Summary analysis of prospective studies has shown that rhBMP-2 is superior to an autograft in obtaining lumbar fusion success [10-12]. Although it remains to be seen whether this is true for maxillofacial reconstructions, studies thus far are promising. In studies comparing iliac crest bone grafts to rhBMP-2 in cleft palate patients and patients undergoing a sinus lift procedure, the groups who received rhBMP-2 performed as well as the autograft groups [13, 14].

In contrast to the rhBMP-2 application with absorbable collagen sponge, transplanted autogenous bone may need to be resorbed or remodelled before fusing. rhBMP-2 may accelerate the creeping substitution of an allograft by stimulating an osteoclastic response with an osteoblastic response. BMP has a role in the regulation of bone turnover via coupled osteoblastic and osteclastic activity and BMP mediated signals are involved in the osteoclastic resorption [15, 16]. 

In 2007, the FDA granted approval of rhBMP-2 (Infuse Bone Graft-Medtronic, Memphis TN^®^) as an alternative to autogenous bone graft sinus augmentation and for localized alveolar ridge augmentations for defects associated with extraction sockets. This approval was based on data from 312 patients enrolled in a total of 5 clinical studies. rhBMP-2 is contraindicated for patients with a known hypersensitivity to rhBMP-2 or bovine type I collagen. It should not be used in the vicinity of a resected tumor, in patients with any active malignancy, in infected sites or pregnant woman [17].

The use of rhBMP-2 delivered to a surgical site in combination with an absorbable collagen sponge has been investigated in preclinical and clinical studies of localized alveolar grafting as well as sinus floor augmentation prior to implants. A pivotal study indicated that the use of rhBMP-2 provides clinical and radiographic results equivalent to those after the use of autogenous grafting [18].

The aim of this work is to investigate the clinical and radiographic long-term results of a hemi-mandibular reconstruction by using rhBMP2, absorbable collagen sponge and titanium mesh.

## CASE REPORT

**Fig. (1):**
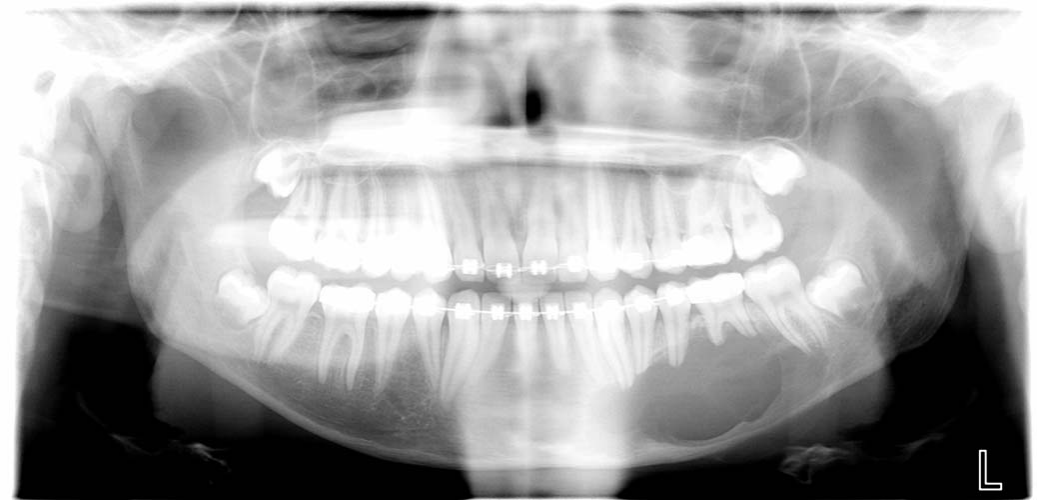
Orthopantomography showed long mandibular circular lesion involving several lower jaw teeth.

**Fig. (2):**
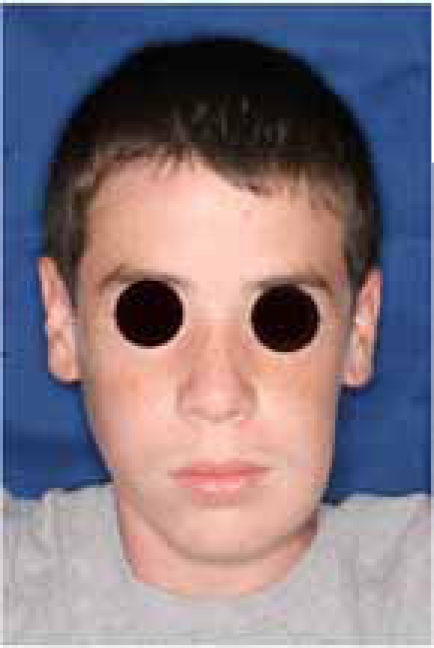
A significant swelling in the left mandible may be appreciated before the tumor resection.

**Fig. (3):**
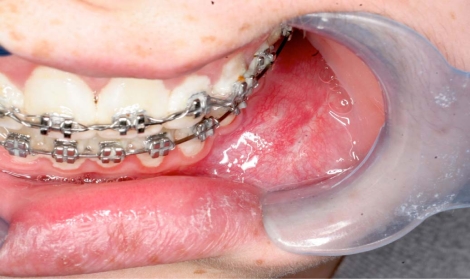
Clinical left mandibular swelling.

An 18 year old patient was undergoing orthodontic treatment by a local dentist. During a visit, the dentist recorded a consistent swelling in the mandibular left corpus. A presumptive diagnosis of vascular malformation was made, and the patient was referred to the author for management. A panorex investigation showed a large tissue mass and extension of the lesion, and a more ominous growth was suspected (Figs.**1**, **2**, **3**). 

**Fig. (4):**
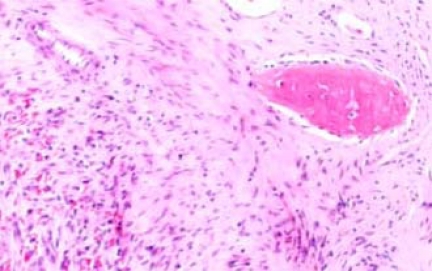
Dentinogenic Ghost Cell Tumor Histological features.

**Fig. (5):**
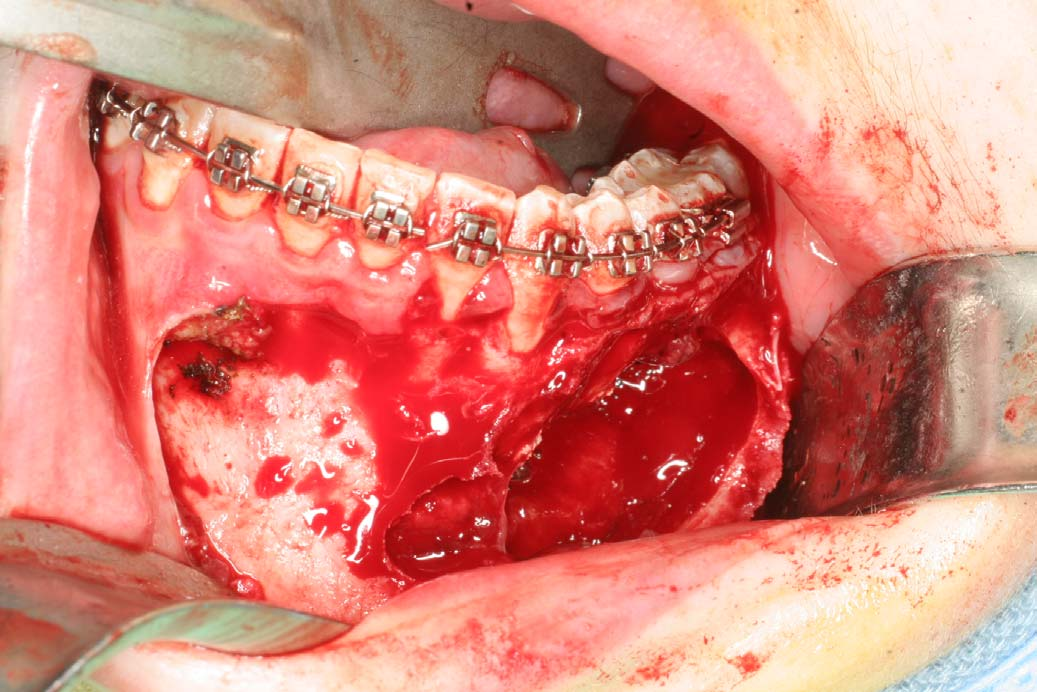
Anatomic tumor extension after the elevated flap.

**Fig. (6):**
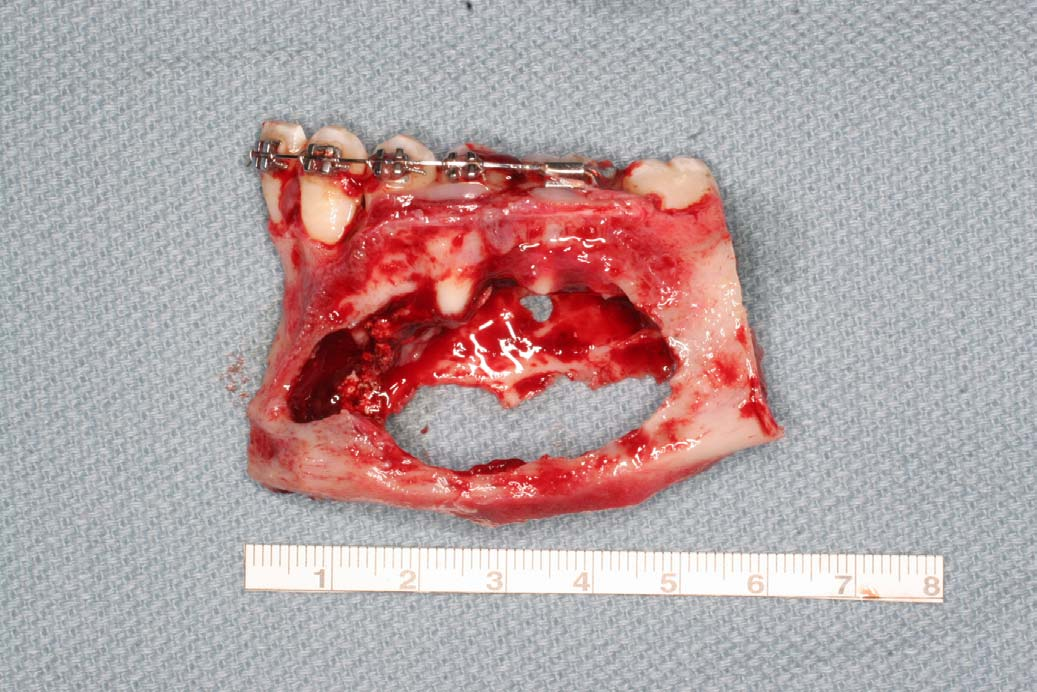
Tumor consistence is clear at the resection time.

**Fig. (7):**
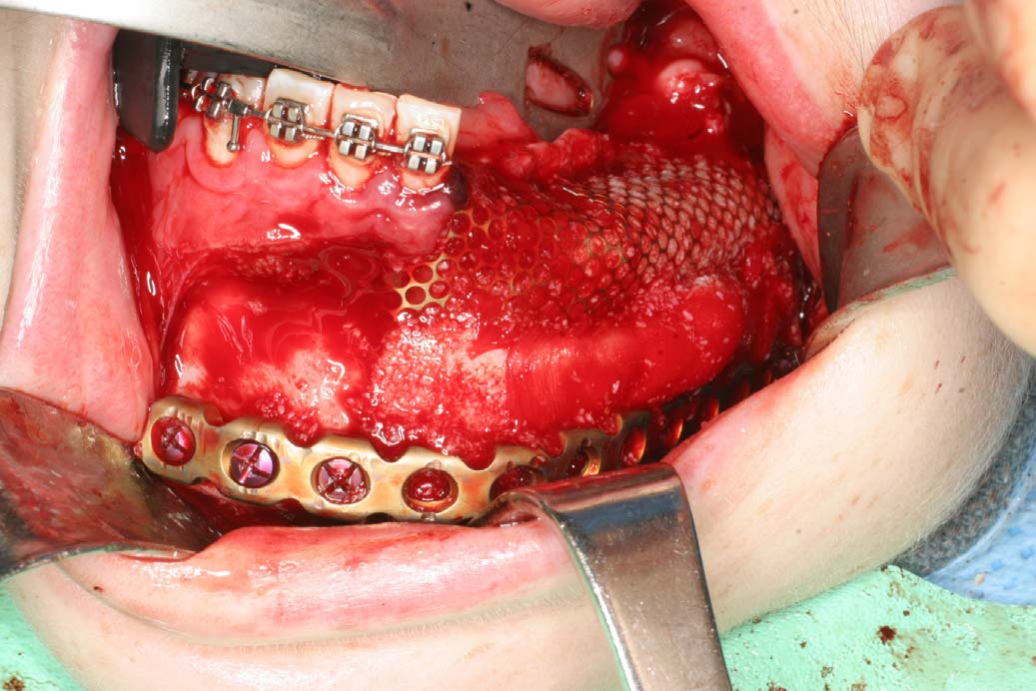
Mandibular reconstruction is performed with low titanium plate, rhBMP-2 and ACS covered by a titanium mesh.

**Fig. (8):**
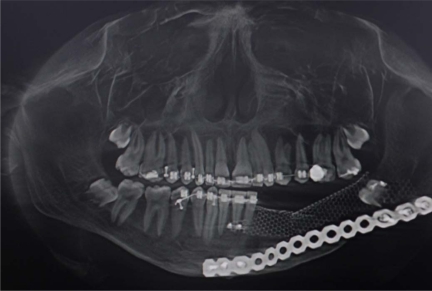
Post op radiographic evaluation.

**Fig. (9):**
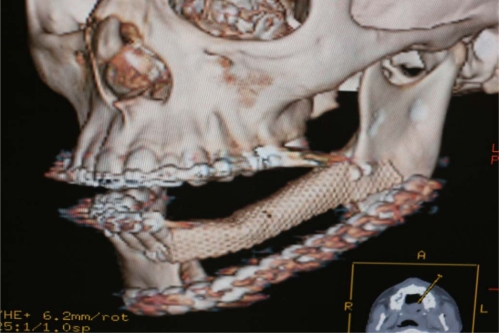
Post op TC evaluation.

**Fig. (10):**
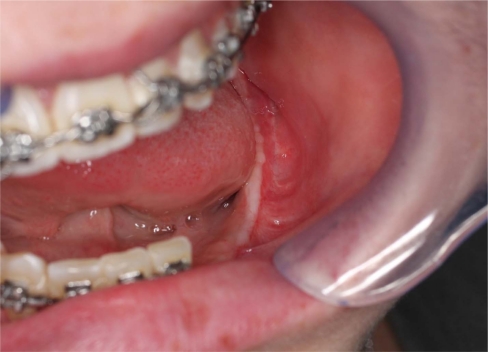
A 3-4 months clinical evaluation. A good soft tissue healing can be appreciated.

**Fig. (11):**
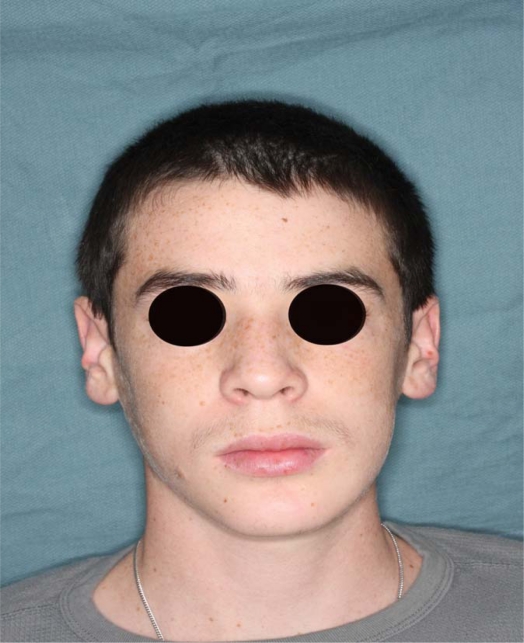
No more swelling is underlined at frontal image 6 months post op.

**Fig. (12):**
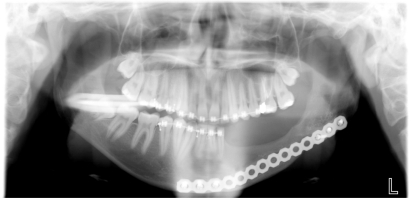
OPT evaluation at 9 month follow up at removing titanium mesh time. Bone regeneration can be appreciated on the left body mandible.

**Fig. (13):**
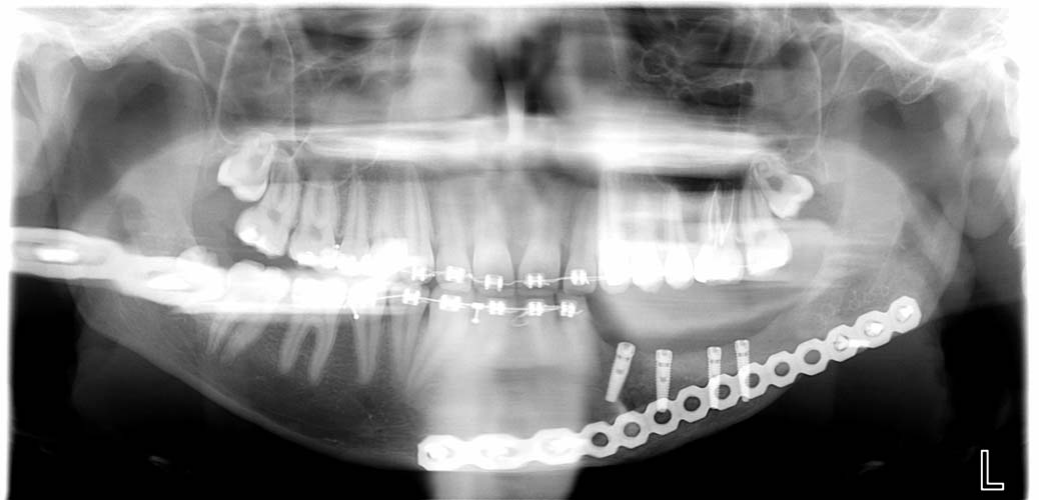
4 dental implants positioned at 9 month follow up after tumor resection in order to reconstitute function and aesthetic of the left mandible area.

A biopsy was taken from the lesion and the histological report showed it to be a Dentinogenic Ghost Cell Tumor (Fig. **4**). The decision was made to manage the local tumor with en bloc resection and immediate reconstruction by using an inferior titanium plate, in order to maintain, without alteration, the mandibular soft tissue space. Moreover, rhBMP2 and absorbable collagen sponge (ACS) were applied inside a titanium mesh (Figs. **5**-**6**). The patient was then placed into a maxillomandibular fixation (MMF) and an inferior 2.4-mm locking reconstruction plate (Synthes, Paoli, PA) was chosen for the mandibular reconstruction. A full-thickness incision was made in the mucosa along the defect. An advantage of placing the incision more buccally is that closure after grafting is much easier. The incision was extended to the bone. Then, subperiosteal dissection was used to adequately expose the entire defect. Broad exposure of the underlying ridge is important as it allows better visualization of the defect as well as freeing up soft tissue for easier closure. Important anatomical landmarks (mental nerve) can be visualized if deemed necessary in order to avoid damage. Once the underlying ridge was exposed, a mesh was countered to correct the defect. It is important to overcorrect by as much as 10-20% as some resorption is expected. Anterior defects require a curvature of the mesh in order to rebuild a natural curve of the arch. The underlying ridge is pierced with a small drill to stimulate bleeding. This accomplishes faster integration of the graft as well as supplying additional stem cells to the area. rhBMP-2 was then placed on the absorbable collagen sponge. A portion of the collagen sponge was then cut into small 2-3 mm pieces and mixed throughout a bone allograft (mineralized or demineralized). Next, the rhBMP-2/absorbable collagen sponge/allograft was placed into the mesh (Fig. **7**). The mesh and graft material were then secured in place with a minimum of two screws but preferably more. Monocortical screws were used in the area of the inferior alveolar nerve in order to avoid nerve damage. TC and OPT post op were performed (Figs. **8**, **9**). The postoperative course was uneventful with the patient doing very well. On examination of the patient at the 3 month follow up, clinical palpation of the mucosa overlying the resected area showed a hard indurated calcifying surface of the regenerated bone. The patient exhibited radiographic evidence of bone formation as early as 3 or 4 months post-operatively, and mandibular continuity was regained as demonstrated both clinically and radiographically (Figs. **10**, **11**). At the 9 month follow up, the titanium mesh was removed and dental implants were placed in position (Figs. **12**, **13**). 

## DISCUSSION

Pre-prosthetic augmentation procedures, including alveolar ridge augmentation, using bone grafts are commonplace. Jovanovic *et al.* performed a histologic study of a canine ridge augmentation with BMP [19]. They found no significant difference between implants with rhBMP-2 induced bone compared to resident bone. rhBMP-2 allows installation, osseointegration, and long term functional loading of dental implants.

According to a study performed by Degidi *et al.*, titanium mesh is helpful in maintaining space with large mandibular and maxillary defects. Moreover, the use of occlusive membranes (resorbable or non resorbable) may prevent vascular in-growth and decrease the available adult stem cells [20]. 

The osteoconductivity capabilities of rhBMP-2 have been widely studied in different bone healing environments. Preclinical and clinical research has demonstrated that rhBMP-2 combined with absorbable collagen sponge can induce new bone formation. rhBMP-2 has been show to heal critical size bone defects in animal models as well as clinical trials [21, 22].

The half-life of rhBMP-2 is only minutes in the bloodstream; thus, it must be administered locally over a period of time to bleeding bone to stimulate bone induction associated with new bone formation. The absorbable, collagen sponge carrier matrix provides a means of delivering rhBMP-2 to the surgical site and retains the growth factor at the site. It has a limited ability to prevent soft tissue prolapse into the defect thus enabling bony vascular growth to occur during rhBMP-2 induced bone formation [23]. The ACS is type I collagen derived from highly purified bovine tendon. The ACS lacks structural stability and is compressed by the soft tissue walls of the defect. Future carriers with more structural stability will aid in maintaining the space for optimal bone formation to occur. The absorbable collagen sponge is susceptible to compression from the overlying tissue. This compression problem has meant that other ways to maintain space should be considered. Options include engineering an alternative carrier, addition of compressive resistant osteoconductive material, supporting the space with a membrane or mesh, or using screws or implants to “tent up” the tissue. Combining the rhBMP-2 with a graft extender may improve the economic feasibility of rhBMP-2 reducing the required protein dose [24].

Both rhBMP-2 and rhBMP-7 have been studied in thousands of patients and tens of thousands of animal with a high safety profile [25]. It is estimated that over 500,000 patients have been treated with rhBMP-2. Low risk, similar to clinical trials leading to approval, can be expected with “on label” use. Transient increases in antibodies to BMPs, developed in 5-10% of patients, does not affect bone healing on first exposure but little is known to-date regarding the effects of multiple exposures. Carreon *et al.* studied patients who were re-exposed to rhBMP for spine surgery [26]. In the ninety-six patients who had at least 2-spine surgeries using rhBMP-2, they found no significant difference in the number of complications between the first and second surgeries. There were no wound problems or allergic reactions among the twelve patients who had a third surgery with rhBMP-2. They concluded that multiple exposures to rhBMP-2 does not increase the risk of wound infections/problems or result in clinically detectable allergic reactions.

When used “off label”, there are possible adverse results that must be balanced against the benefit of using rhBMP-2. Changing the recommended concentration resulted in inconsistent bone formation. If a higher concentration is used, local edema or fluid connection may occur as has been reported in some cases of anterior cervical discectomy and fusion using BMP instead of autogenous bone [27]. Adverse facial edema has also been noted when rhBMP-2 was used for cranial reconstruction for craniosynostosis [28]. Because BMPs can accelerate bone resorption in addition to bone formation, using more resorbable carriers which have not been carefully tested with them, may result in accelerated resorption of the bone. As with any new technology, concerns over additional costs of such interventions should be considered. As discussed by Kuklo et al. there is a compelling argument for the continued use of such technologies, as the primary outcome measures of union, rate of infection, and reoperation were all improved with rhBMP-2 in a large number of patients [29].

Despite the extent of bone formation, there has not been any evidence of bone formation extending beyond the boundaries of the defect. The process of induced bone formation is a controlled response to highly concentrated levels of rhBMP-2. This bone inducing protein is normally present endogenously in the body and it is likely that normal growth regulating genes control the growth process and prevent over-growth.

Even if the use of rhBMP-2 in oral surgery seems to give predictable and long term results as shown from the large number of animal studies and clinical trials, several questions are still unanswered such as:

- What is the ideal grafting material to combine with BMPs to enhance bone formation in a specific defect?
- Does the addition of BMPs to autogenous bone improve the “gold standard”’
- Will alloplastic, allogenic, or xenogenic graft material in combination with rhBMP-2 prove to be superior than rhBMP-2/ACS alone?
- Are there other cytokines that will enhance the activity of BMPs?

As rhBMP-2 is osteoinductive and allografts are osteoconductive, it appears logical to combine the two in an effort to enhance the amount and rate of bone formation. Future studies will help to shed light on these questions as we continue to strive to improve our understanding of bone healing.

## CONCLUSION

The published literature suggests that rhBMP-2 is clinically effective in treatment of critical size defects in both extremities as well as in the maxillofacial region. Moreover, the excellent results of this case report seems to support the ability of rhBMP-2 to predictably induce new bone formation at the implantation site. Further study will continue to support this conclusion and offer definitive proof that rhBMP-2 & ACS can be a safe alternative to the harvesting of autograft in maxillofacial reconstruction just as it has been in orthopaedic applications. rhBMP-2 repair has significant advantages for the patient: large bone defects do not need non-oral donor sites or long surgical procedures. In the presented case, the possibility of using rhBMP-2 significantly reduced patient pain and discomfort.

Other advantages include shorter stay in hospital, avoidance of gait and sensory disturbance, as well as a decrease in possible infection and also the size of the scar formation area. Surgical time is reduced since the surgeon does not have to harvest autogenous bone from a secondary site. 

In the future, the use of exogenous cytokines, particularly those in the BMPs series, will become common and the regeneration of osseous defects will likely be carried out as a clinical outpatient procedure.

## References

[1]  Wozney JM (2002). Overview of bone morphogenic proteins. Spine.

[2]  Urist MR (1965). Bone formation by autoinduction. Science.

[3]  Ahlmann E, Patzakis M, Roidis N, Shepherd L, Holtom P (2002). Comparison
of anterior and posterior iliac crest bone grafts in terms of
harvest-site morbidity and functional outcomes. J Bone Joint Surg.

[4]  Herford AS, Boyne PJ, Williams RP (2007). Clinical applications of
rhBMP-2 in maxillofacial surgery. CDA Journal.

[5]  Warnke PH, Springer IN, Wiltfang J (2004). Growth and transplantation
of a custom vascularized bone graft in a man. Lancet.

[6]  Arrington ED, Smith WJ, Chambers HG, Bucknell AL, Davino 
NA (1996). Complications of iliac crest graft harvesting. Clin Orthop.

[7]  Banwart JC, Asher MA, Hassanein RS (1995). Iliac crest bone graft harvest
donor site morbidity a statistical evaluation. Spine.

[8]  Goulet JA, Senunas LE, DeSilva GL (1997). Autogenous iliac crest bone
graft. Clin Orthop.

[9]  Burkus JK, Heim SE, Gornet MF, Zdeblick TA (2003). Is infuse bone
grafts superior to autograft? an integrated analysis od clinical trials
using the LT-CAGE lumbar tapered device. J Spinal Discord Tech.

[10]  Carragee EJ, Mitsunaga KA, Hurwitz EL, Scuderi GJ (2011). Retrograde
ejaculation after anterior lumbar interbody fusion using rhBMP-2 a
cohort controlled study. Spine J.

[11]  Boden S, Zdelblick T, Sandu H, Heim SE (2000). The use of rhbmp-2 in
interbody fusion cages. Spine.

[12]  Burkus J, Gornet M, Dickman C, Zdeblick TA (2002). Anterior lumbar
interbody fusion using rhbmp-2 with tapered interbody cages. J
Spin Discord Tech.

[13]  Herford AS, Boyne PJ, Rawson R, Williams RP (2007). Bone morphogenetic
protein-induced repair of the premaxillary cleft. J Oral Maxillofac
Surg.

[14]  Alonso N, Tanikawa DY, Rida FS, Canan L Jr, Ozawa TO, Rocha 
DL (2010). Evaluation of maxillary alveolar reconstruction using a resorbable
collagen sponge with recombinant human bone morphogenetic
protein-2 in cleft lip and palate patients. Tissue Eng Part C Methods.

[15]  Poyton AR, Lane JM (2002). Safety profile for the clinical use of bone
morphogenic proteins in the spine. Spine.

[16]  Itoh K, Udagawa N, Katagiri T (2001). Bone morphogenic protein-2
stimulates osteoclast differentiation and survival supported by receptor
activator of nuclear factor-kB ligand. Endocrinology.

[17]  Triplett RG, Nevins M, Marx R (2009). Pivotal randomized parallel
evaluation of recombinant human bone morphogenetic protein-
2/absorbable collagen sponge and autogenous bone graft for maxillary
sinus floor augmentation. J Oral Maxillofac Surg.

[18]  Carragee EJ, Hurwitz EL, Weiner BK (2011). A critical review of recombinant
human bone morphogenetic protein-2 trials in spinal surgery
emerging safety concerns and lessons learned. Spine J.

[19]  Jovanovic SA, Hunt DR, Bernard GW (2003). Long term functional
loading of dental implants in rhBMP-2 induced bone. a histological
study in the canine ridge augmentation model. Clin Oral Implants
Res.

[20]  Degidi M, Scarano A, Piattelli A (2003). Regeneration of the alveolar crest
using titanium micromesh with autologous bone and a resorbable
membrane. J Oral Implantol.

[21]  Herford AS, Cicciù M (2010). Recombinant human bone morphogenetic
protein type 2 jaw reconstruction in patients affected by giant cell
tumor. J Craniofac Surg.

[22]  Boyne PJ (1996). Animal studies of application of rhBMP-2 in maxillofacial
reconstruction. Bone.

[23]  Boyne PJ, Lilly LC, Marx RE (2005). De novo bone induction by
recombinant human bone morphogenetic protein-2 (rhBMP-2) in
maxillary sinus floor augmentation. J Oral Maxillofac Surg.

[24]  Akamaru T, Suh D, Boden S, Kim HS, Minamide A, Louis-Ugbo J (2003). Simple carrier matrix modifications can enhance delivery of recombinant
human bone morphogenetic protein-2 for posterolateral
spine fusion. Spine.

[25]  Boden SD (2005). The ABCs of BMPs. Orthopaed Nurs.

[26]  Carreon LY, Glassman SD, Brock DC, Dimar JR, Puno RM, Campbell MJ (2008). Adverse events in patients re-exposed to bone
morphogenic protein for spine surgery. Spine.

[27]  Poynton AR, Tomin E, Cornwall GB, Zheng F, Lane JM (2002). Resorbable posterolateral graft containment in a rabbit spinal fusion model. Orthopedics.

[28]  Shah MM, Smyth MD, Woo AS (2008). Adverse facial edema associated
with off-label use of recombinant human bone morphogenic protein-
2 in cranial reconstruction for craniosynostosis. J Neurosurg Pediatr.

[29]  Kuklo TR, Groth AT, Anderson RC, Frisch HM, Islinger RB (2008). Recombinant
human bone morphogenetic protein-2 for grade III open
segmental tibial fractures from combat injuries in Iraq. J Bone Joint Surg Br.

